# Supplementary material for: A simulation of older adults’ associative memory deficit using structural process interference in young adults
Source: PLoS One. 2021 Nov 18;16(11):e0258574. doi: 10.1371/journal.pone.0258574 (PMC8601526; doi:10.1371/journal.pone.0258574)
Supplement: Attachment — Submitted filename: response to reviewers 9.2.docx [file pone.0258574.s003.docx]

PONE-D-20-30627
A simulation of older adults’ associative memory deficit
using structural process interference in young adults
PLOS ONE

Dear Dr. Oscar-Strom,

Thank you for submitting your manuscript to PLOS ONE. After careful consideration, we feel that it has merit but does not meet PLOS ONE’s publication criteria as it currently stands. In particular, the research question needs to be clearly motivated and discussed in the context of the relevant literature, and there are open questions regarding the methods and results. Therefore, we invite you to submit a substantially revised version of the manuscript that addresses the points raised during the review process.

Editor Comments:

Two expert reviewers assessed this work, and they largely agree that substantial revisions are required to make this manuscript acceptable for publication. Both reviewers agree that the introduction and discussion cover aspects (e.g. neural processes) that are speculative and irrelevant to the research question at hand, while omitting more relevant literature. These sections should thus be rewritten to reflect the relevant background, and appropriately motivate the current experiments. **It is also important to clarify the choice of task, and whether the intention was to use a task (NBL) with a working memory (WM) component compared to the other tasks not involving WM.**

The reviewers also comment that insufficient information is provided regarding participants and tasks.

The other comments mainly concern the statistical analyses, where additional information is required to aid interpretation of the results.

Please note that while novelty is not a publication criterion for PLOS ONE, any published work need to be methodologically sound, and the conclusions need to be supported by the data.

Please submit your revised manuscript by Jan 09 2021 11:59PM. If you will need more time than this to complete your revisions, please reply to this message or contact the journal office at plosone@plos.org. When you're ready to submit your revision, log on to https://www.editorialmanager.com/pone/ and select the 'Submissions Needing Revision' folder to locate your manuscript file.

Please include the following items when submitting your revised manuscript:

- A rebuttal letter that responds to each point raised by the academic editor and reviewer(s). You should upload this letter as a separate file labeled 'Response to Reviewers'.
- A marked-up copy of your manuscript that highlights changes made to the original version. You should upload this as a separate file labeled 'Revised Manuscript with Track Changes'.
- An unmarked version of your revised paper without tracked changes. You should upload this as a separate file labeled 'Manuscript'.

If you would like to make changes to your financial disclosure, please include your updated statement in your cover letter. Guidelines for resubmitting your figure files are available below the reviewer comments at the end of this letter.

If applicable, we recommend that you deposit your laboratory protocols in protocols.io to enhance the reproducibility of your results. Protocols.io assigns your protocol its own identifier (DOI) so that it can be cited independently in the future. For instructions see: <http://journals.plos.org/plosone/s/submission-guidelines#loc-laboratory-protocols>

We look forward to receiving your revised manuscript.

Kind regards,

Maria Wimber
Academic Editor
PLOS ONE

Journal Requirements:

When submitting your revision, we need you to address these additional requirements.

1. Please ensure that your manuscript meets PLOS ONE's style requirements, including those for file naming. The PLOS ONE style templates can be found at

https://journals.plos.org/plosone/s/file?id=wjVg/PLOSOne_formatting_sample_main_body.pdf and

https://journals.plos.org/plosone/s/file?id=ba62/PLOSOne_formatting_sample_title_authors_affiliations.pdf

2. Our internal editors have looked over your manuscript and determined that it is within the scope of our Cognitive Developmental Psychology Call for Papers. The Collection will encompass a diverse range of research articles in developmental psychology, including early cognitive development, language development, atypical development, cognitive processing across the lifespan, among others, with an emphasis on transparent and reproducible reporting practices.  Additional information can be found on our announcement page: **https://collections.plos.org/s/cognitive-psychology.**  If you would like your manuscript to be considered for this collection, please let us know in your cover letter and we will ensure that your paper is treated as if you were responding to this call. Please note that being considered for the Collection does not require an additional peer review beyond the journal’s standard process and will not delay the publication of your manuscript if it is accepted by PLOS ONE. If you would prefer to remove your manuscript from collection consideration, please specify this in the cover letter.

3. Please include captions for your Supporting Information files at the end of your manuscript, and update any in-text citations to match accordingly. Please see our Supporting Information guidelines for more information: [http://journals.plos.org/plosone/s/supporting-information](about:blank).

[Note: HTML markup is below. Please do not edit.]

Reviewers' comments:

Reviewer's Responses to Questions

**Comments to the Author**

1. Is the manuscript technically sound, and do the data support the conclusions?

The manuscript must describe a technically sound piece of scientific research with data that supports the conclusions. Experiments must have been conducted rigorously, with appropriate controls, replication, and sample sizes. The conclusions must be drawn appropriately based on the data presented.

Reviewer #1: No

Reviewer #2: Partly

2. Has the statistical analysis been performed appropriately and rigorously?

Reviewer #1: Yes

Reviewer #2: Yes

3. Have the authors made all data underlying the findings in their manuscript fully available?

The [PLOS Data policy](http://www.plosone.org/static/policies.action#sharing) requires authors to make all data underlying the findings described in their manuscript fully available without restriction, with rare exception (please refer to the Data Availability Statement in the manuscript PDF file). The data should be provided as part of the manuscript or its supporting information, or deposited to a public repository. For example, in addition to summary statistics, the data points behind means, medians and variance measures should be available. If there are restrictions on publicly sharing data—e.g. participant privacy or use of data from a third party—those must be specified.

Reviewer #1: Yes

Reviewer #2: Yes

4. Is the manuscript presented in an intelligible fashion and written in standard English?

PLOS ONE does not copyedit accepted manuscripts, so the language in submitted articles must be clear, correct, and unambiguous. Any typographical or grammatical errors should be corrected at revision, so please note any specific errors here.

Reviewer #1: No

Reviewer #2: Yes

5. Review Comments to the Author

Please use the space provided to explain your answers to the questions above. You may also include additional comments for the author, including concerns about dual publication, research ethics, or publication ethics. (Please upload your review as an attachment if it exceeds 20,000 characters)

Reviewer #1: The paper proposed here explores the impact of divided attention at encoding on subsequent item and associative memory in young adults, across two experiments that vary on the task used to create divided attention (Experiment 1 using N-back like (NBL) task vs. parity decision and Experiment 2 using NBL task vs. a visuo-spatial decision task), with the aim to simulate the age-related associative memory deficit in young subjects. I have several major concerns about the paper in its current form, that make me lean towards recommending its rejection. I found the introduction to be incomplete. The use of references is not specific enough, and references in general are not sufficiently provided given the wide literature existing on the topic of age-related associative memory decline. Some details that are irrelevant for the current study are provided (e.g., the associative index, that is then not used in the analyses, or brain regions associated with the cognitive mechanisms while the study does not include neuroimaging data), while I found that the details necessary to understand the rationale of the current study were lacking (i.e (p.2), why the previous studies manipulating divided attention to simulate the associative decline weren’t good enough to interfere with the specific associative binding process; this needs to be much more developed in my opinion).

Thanks for your comment. We extended the introduction so now it is based on more essential studies on this topic. We elaborated more on the details needed to explain our rationale, and we specifically addressed that previous studies have used CRT secondary tasks which require information that already exists in the LTM. CRT secondary tasks were not involved WM updating or other demanding process that required WM and therefore not interfere to the associative binding in working Memory. Our secondary task is a continuous WM task that requires updating and maintenance, so the participants are required to divide their attention throughout the time of performing the task. Regarding the associative index, it was calculated and presented in figure 4 and elaborated on in the discussion (PP. 17). Indeed, the brain regions are less relevant for the introduction of this paper, as this is not a neuroimaging study. However, we still addressed it but minimized it in the discussion, and based on known literature as an additional interpretation to our results.

The methods should give more information concerning the participants: was the sample size determined by a-priori power analysis?

Thanks for your comment. The number of participants used for the current study corresponds to other scientific work in the field (for example: Naveh-Benjamin, Craik, Guez, and Kreuger, 2005 [with 16 participants for each condition/group]; Naveh-Benjamin, Guez, & Marom, 2003 [experiment 2]).

Calculation of estimated sample size using G*Power software, version 3.1.9.2 yielded 28 participants. (Taking the critical interaction between group/task and tests under DA condition, with moderate parameters of effect-size = 0.3; Anova: repeated measures, within-between interaction). For the main effects only, the appropriate sample size is between 10-14 participants.

Were the participants screened for any neurological or psychiatric history? As participants were divided into two groups, were the groups matched in terms of age and education? Was the study approved by an Ethics Committee?

The participants section is now extended, see pp. 6 for experiment 1. Also at pp. 13 for experiment 2.

Regarding the way participants were divided into groups, Participants were randomly assigned to each group. See in the procedures section pp. 9 for experiment 1 and pp. 13 for experiment 2.

The study was approved by the local institutional review board of Achva Academic College. All participants gave their written informed consent for study participation.

Concerning the methods still, I am not convinced by the choice of the secondary tasks and what they allow to show evidence for, and I did not find the information put clearly in the introduction or hypotheses. From my understanding, only the NBL task requires working memory, while the two other secondary tasks to which it is compared do not; they are parity and visuo-spatial judgment tasks. So, if I understood correctly, the task comparison implies working memory task vs. visuo-spatial or parity decision, non-working memory tasks. **I think this should be put very clearly to make it clear to the reader what are the stakes of such comparison, and what they are not**.

You are right. We have clarified the differences between the secondary tasks in the introduction. In the task analysis we emphasized that the VS and parity judgement tasks are CRT tasks that rely on information from the LTM. Thus, those tasks do not involve WM updating or other demanding process that requires WM and therefore do not interfere with the associative binding in working Memory. However, the NBL task requires updating and maintenance and therefore throughout the performance of the task, attention is divided through all the experiment and does not allow creating the association in WM.

I have one problem with the results where the attention (full or divided; within subject factor) and task (NBL or alternative secondary task; across subjects factor) variables are analyzed in a factorial design. Caution should be given to indicate whether there is, or not, a difference between groups in the full attention condition (e.g., according to the attention x task interaction). Indeed, one would expect to observe no “group-task” difference in the full attention condition, as there is no secondary task to perform in that condition. Showing this absence of difference would help ensuring that the effect of task is indeed caused by the actual secondary task, when there is one, and not group difference in “baseline” (full attention) memory. Yet, these results are not reported, but there is a main effect of task, and thus, of group.

Thank you for your comment. We have now added a comparison between groups at the full attention condition to test possible differences at the baseline measures. In both experiments, pre-analysis, at the full attention condition (baseline), showed no difference between groups on item and associative recognition. These results strengthen our interpretation of the observed differences on the experimental condition being due to the experimental manipulation.

2X2 analysis between group and test in FA condition (experiment 1) yield no significant effect for group [*F* (1, 32) = 0.72, *p* = 0.40, η2 = 0.01] nor for the interaction between test and group [*F* (1, 32) = 1.31, *p* = 0.26, η2 = 0.04].

The same was found for experiment 2. No significant effect for group [*F* (1, 30) = 1.10, *p* = 0.30, η2 = 0.03] nor for the interaction between test and group [*F* (1, 30) = 0.00, *p* = 0.99, η2 = 0.00].

This is now reported at page 10 and page 14 respectively.

In addition, I have a great concern about the discussion part, which I found highly speculative in several aspects. Among my concerns are that many of the conclusions drawn are not supported by the data as they imply brain regions, but also are extended to other type of populations than older adults whose associative decline was simulated, such as patients with post-traumatic stress disorder.

Thanks for your comment. PTSD was removed from the paper. Neuroimaging was removed from the introduction and minimized in the discussion. It is discussed as an optional interpretation of the results.

Finally, I found that the paper would highly benefit from some English editing.

We sent the article to English editing.

Reviewer #2: With two behavioral experiments, the present study compared item and associative memory performance for word pairs encoded with full vs. divided attention. In each experiment, participants encoded half of the stimuli with full attention, and the other half of the stimuli while also performing one of two secondary tasks (N-back like task and parity task in experiment 1, N-back like task and visuospatial task in experiment 2). Findings indicated that the N-back like task affected associative memory to a larger extent than item memory. By contrast, the other two tasks showed comparable influences on item and associative memory performance. I find these findings are interesting, although a few questions and comments need to be addressed.

1. From an overall point of view, the current study provides behavioral evidence for the shared cognitive processes recruited by NBL and associative encoding. There is a decently sized discussion of the potential neural mechanisms contributing to these processes in both the intro and discussion, which I’m not sure are clearly tied to the predictions/results, given that this is a behavioral study, and no direct comparison was made between the neural correlates of NBL and associative encoding. I would suggest the authors to focus more on the implications of the findings from a cognitive perspective.

Thanks for your comment. This is a major point. We have now revised the paper focusing more on the behavioral data and cognitive processes and minimized the neural mechanisms interpretation.

Methods:
2. Were the participants randomly assigned to the groups with different secondary tasks?

Yes. Participants were randomly assigned to each group. See pp. 9 for experiment 1; and pp. 13 for experiment 2.

3. Please clarify the instruction for the FA condition.

In the full attention (FA) condition participants performed the memory task (learning phase and the two recognition tests (item and associative recognition)) the same as in the DA condition, but without the concurrent secondary task (either NBL, parity-task, or VS task). To clarify this in the paper we have added sub-title for "memory learning phase" see page 7 and sub-title for "memory test phase" (include item and associative recognition) see pp.8. Additionally, we edited the method section that will be more understanding.

Results:
4. Across the two experiments, the group x test x attention interaction term from the initial 2 (task) x 2 (test) x 2 (attention) ANOVA was not significant. Therefore, it is not justified to examine the attention x test interaction for each group.

Thanks for your comment. We think there are two ways to present the results. One way is to test only the 2X2 interaction between group and test at the FA condition to show that there is no difference between the groups at baseline, and then move to the critical 2X2 interaction group and test under DA condition. The other way is to present the results with a full factorial analysis. Following your comment, we now have added the initial analysis in both experiments, at the FA condition. No difference between groups on item and associative recognition was observed. These results strengthen our interpretation regarding the observed differences on the experimental condition being due to the experimental manipulation.

2X2 analysis between group and test in FA condition (experiment 1) yield present no significant effect for group [*F* (1, 32) = 0.72, *p* = 0.40, η2 = 0.01] nor for the interaction between test and group [*F* (1, 32) = 1.31, *p* = 0.26, η2 = 0.04].

The same was found for experiment 2. no significant effect for group [*F* (1, 30) = 1.10, *p* = 0.30, η2 = 0.03] nor for the interaction between test and group [*F* (1, 30) = 0.00, *p* = 0.99, η2 = 0.00]. This is now reported at page 10 and page 14 respectively].

We now present the above "baseline" comparisons and continue analysed the full factorial design, which presents the whole picture with the relevant planned-comparison. If necessary, we can present the critical 2x2 analysis (at the DA condition) after the 2x2 analysis for the baseline FA condition.

Furthermore, significant ANOVA results are not a pre-condition for performing focused contrasts (see Rosnow & Rosenthal, 1995, p. 4). Although the three-way interaction in the full factorial analysis was not significant, we performed our planned comparisons to test the interaction between *group* and *test* separately for each attention condition. We have added this reference and note it in a comment for the reader at pp. 10.

5. In p9, paragraph 1: the author mentioned larger associative deficit in the DA condition than the FA condition. Was the deficit measured as ADI? Please clarify the operational definition of associative deficit in the method section.

Thanks for your comment. Associative-deficit refers to memory performance decrease from item to associative recognition (this is the meaning in the mentioned paragraph, and it is clearly demonstrated on figure 2). The associative-deficit index (ADI) is a simple calculation of the difference between item recognition and associative recognition performance, and we used it in the additional analysis across experiments and condition, see paragraph 2 in the discussion.

Therefore, we have now added to the scoring section a sentence "In accordance, associative-deficit refers to the difference between item recognition and associative recognition performance” and refer to the ADI and its meaning at the discussion.

Also, it would be informative to show whether the associative deficit (relative to the FA condition) was larger in the NBL task compared to the other two tasks.

We have now added a final analysis taking the overall data from the two experiments. ADI was the dependent measure. Because no differences were observed in ADI in the FA conditions (F < 1) we put them together (see figure 4). Then we used one-way ANOVA to test difference in ADI between FA, Non-WM tasks (parity and VS), and N-back task.

To directly compare the associative-deficit across experiments and condition we used Associative-deficit index (ADI - calculation of the difference between item recognition and associative recognition performance). Four attention conditions were compared in a one-way Anova; FA – the overall participant under full attention; DA-parity; DA-VS; DA-N-back. Results are presented in figure 4.

These results emphasize our hypothesis that only N-back task simulates associative-deficit in young adults. We can draw from this observation that only tasks that employed WM-updating, but not a dual-task that competes with attentional resources, can affect associative binding. This simulation suggests that older adults suffer from damage of the same cognitive process/brain circle involved in a WM-update.

6. Table 1&2: It would be helpful to add one row for each memory type to present the hits-FAs. It seems that in the FA condition, the group with NBL task in both experiments had lower associative memory performance compared to the other groups. Have the authors compared memory performance between the task groups?

Done. We have added another two rows with the percentage HITs minus percentage FAs. For each experiment (see tables 1 & 2).

It seems that in the FA condition, the group with NBL task in both experiments had lower associative memory performance compared to the other groups. Have the authors compared memory performance between the task groups? Is it possible that poor associative memory in the NBL group drove the significant attention x test interaction in this group?

You are right, there is about a 10% decrease in the performance in the FA condition of the NBL group compared to the other group. As mentioned, the assignment to the groups was random. Furthermore, comparing the FA (baseline) condition between groups yielded no significant effect or interaction. These results strengthen our interpretation regarding the observed differences in the experimental condition being due to the experimental manipulation.

2X2 analysis between group and test in FA condition (experiment 1) yield present no significant effect for group [*F* (1, 32) = 0.72, *p* = 0.40, η2 = 0.01] nor for the interaction between test and group [*F* (1, 32) = 1.31, *p* = 0.26, η2 = 0.04].

The same was found for experiment 2. no significant effect for group [*F* (1, 30) = 1.10, *p* = 0.30, η2 = 0.03] nor for the interaction between test and group [*F* (1, 30) = 0.00, *p* = 0.99, η2 = 0.00].

This is now reported at page 10 and page 14 respectively].

Discussion:
7. p13, paragraph 2: Because this is a behavioral study on young adults only, it is difficult to draw a conclusion that circuit brain areas that are involved in the NBL tasks are impaired in older adults. Is there any behavioral evidence linking performance on N-back task to the performance of associative memory?

We have removed the circuit brain areas from the introduction. Additionally, it is no longer one the main conclusions of this article. Regarding the behavioural data linking performance on N-back task to the performance of associative memory, so far, in the literature some studies found evidence, and some did not. Therefore, it is still inconclusive. See Lugtmeijer,Haan, & Kessels, (2019).

8. p14, paragraph 2: I’m not sure if the findings from PTSD and ASD patients are relevant, given that the main focus of the current study was to simulate age-related associative deficit in young adults.

The PTSD and ASD part in the discussion was removed from the paper.

9. p14, paragraph 3: Could the authors further discuss the study of Hara et al. (2015)? I think this study provide consistent findings with the current study that WM processing is a key component contributing to associative encoding.

We have now discussed the Hara et al (2015) study in the introduction and discussion in specific, and generally on more relevant studies that showed that a reduction in WM resources is related to associative-deficit in older adults.

6. PLOS authors have the option to publish the peer review history of their article ([what does this mean?](https://journals.plos.org/plosone/s/editorial-and-peer-review-process#loc-peer-review-history)). If published, this will include your full peer review and any attached files.


If you choose “no”, your identity will remain anonymous but your review may still be made public.


**Do you want your identity to be public for this peer review?** For information about this choice, including consent withdrawal, please see our [Privacy Policy](https://www.plos.org/privacy-policy).

Reviewer #1: **Yes:** Emma Delhaye

Reviewer #2: No

[NOTE: If reviewer comments were submitted as an attachment file, they will be attached to this email and accessible via the submission site. Please log into your account, locate the manuscript record, and check for the action link "View Attachments". If this link does not appear, there are no attachment files.]

While revising your submission, please upload your figure files to the Preflight Analysis and Conversion Engine (PACE) digital diagnostic tool, <https://pacev2.apexcovantage.com/>. PACE helps ensure that figures meet PLOS requirements. To use PACE, you must first register as a user. Registration is free. Then, login and navigate to the UPLOAD tab, where you will find detailed instructions on how to use the tool. If you encounter any issues or have any questions when using PACE, please email PLOS at [figures@plos.org](mailto:figures@plos.org). Please note that Supporting Information files do not need this step.
