# Supplementary material for: A simulation of older adults’ associative memory deficit using structural process interference in young adults
Source: PLoS One. 2021 Nov 18;16(11):e0258574. doi: 10.1371/journal.pone.0258574 (PMC8601526; doi:10.1371/journal.pone.0258574)
Supplement: Attachment — Submitted filename: respones R2 24.4 finalSD.docx [file pone.0258574.s004.docx]

Please use the space provided to explain your answers to the questions above. You may also

include additional comments for the author, including concerns about dual publication, research ethics, or publication ethics. (Please upload your review as an attachment if it exceeds 20,000 characters)

Reviewer #1: I reviewed the manuscript of the paper « A simulation of older adults’ associative memory deficit using structural process interference in young adults”. This paper intends to simulate the age-related associative memory deficit in young adults through divided attention during encoding, with the hypothesis that such deficit can be simulated specifically by a working memory task requiring memory updating, but not using other non-working memory tasks. The results support this hypothesis as the associative memory decline in young adults is specifically shown following the working memory N-back like while it is not the case following two other non-working memory tasks varying in terms of resource demands.
While I think that the study is interesting and could make a nice contribution to the literature, I have several concerns about the manuscript.

First, I really think that the manuscript would highly benefit from English editing as many sentences seem grammatically incorrect, which makes the reading laborious.

We have proofread and edited the manuscript again in close cooperation with an experienced professional editor of scientific articles. We have rewritten some of the longer and difficult sentences.

Introduction
p.6, lines 139-141: “As mentioned above, the CRT task does not involve the binding mechanism in WM but rather relies on retrieval from the LTM” (same idea also appear on p. 8, lines 168-170). Does this imply that the NBL task involves the binding mechanism in WM? And if yes, in what way exactly? Also, it is not clear to me in what way both CRT tasks rely on the retrieval from LTM. Could the authors be more precise on that point?

It is an interesting point. The NBL task does involve the binding mechanism. This WM binding is part of the task demand. Each presented stimulus is dependent on the participant holding three previous stimuli in their WM and updating each of them to previous stimuli. This process involved relational analysis between a sequence of stimuli and in this sense can be defined as WM binding. The NBL task contrasts to the parity and VS tasks in which each stimuli response is independent of previous stimuli.

Regarding parity, in our task analysis, the decision of whether a number is odd or even relies on information that already exists in LTM (now added in page 5 line 110). Regarding VS, this kind of task requires only shallow perceptual analysis and therefore does not require retrieval from LTM (now added in page 5 line 113).

Methods
p. 9: it is not clear to me if there was a memory test after each list, or if the memory test took place after the 4th list. If it took place after the 4th list, it is not clear to me why each list was followed by a distracting phase. Also, did the memory test mix items from the FA and from the DA conditions?

1. Memory test was taken after learning each list (after an interpolated/distracting task of counting backward).
2. The attention factor was between lists, ie each list was presented either under FA or DA conditions.

This is now clearer in the method section see page 8 line 163; we added: "Then, after the distracting task, two memory tests as described below (item test and association test) were commenced."

p. 10, lines 228-231: “Of these, eight were target words which were selected from the studied pairs (1 from each pair) and eight words were distractors. The distractors were eighteen words with the same characteristics as the target words, except that they had not appeared in the study phase.” Was it eight or eighteen distractors?
Thank you for your comment and for bringing this to our attention. There were eight distractors, not eighteen. It is now corrected in the manuscript.

Results
Please homogenize the variable names, as they are sometimes called “group” and sometimes called “task”, sometimes “task-group”.

DONE. It is now corrected in the manuscript to “task-group” throughout the manuscript.

Discussion
p. 20-21: I think results reports would fit better in the result section than in the discussion part.

DONE. We have added a new section before the general discussion *Further analysis across experiments* see page 16 line 336.

Reviewer #2: The authors have addressed most of my prior questions. Now I only have some minor comments for the revised manuscript.
First, please incorporate the results about ADI (in discussion) into the results section.

DONE. We have added a new section before the general discussion *Further analysis across experiments* see page 16 line 336.

Second, the authors mentioned the interaction of MTL/H and mPFC in supporting memory retrieval in the discussion. Is there any evidence suggesting mPFC is involved in NBL-like working memory? Again, I think the discussion of the specific brain regions is not highly relevant to the present study.

Thank you for your comment. Based upon it, we discussed this issue and accepted that this part should be removed from the manuscript.

Third, as the authors described now, associative deficit is defined as the decreased performance from item memory to associative memory, which is different from the original definition by Naveh-Benjamin (2000). If the authors stick to the current definition (which means AD is present whenever an effect of test is identified), then please make sure to use this term properly throughout the manuscript. For example, please clarify the sentence in line 614: previous associative deficit studies reported on minimum memory deficit during retrieval compared to encoding.

Thanks for your comments.

1. You are right. Naveh-Benjamin refers to Age associative deficit as expressed by a larger effect of age on memory for associative information than for item information. Thus, in order to identify a specific associative deficit it must be compared to item recognition which is exactly our definition of associative deficit.

In our work, we define Associative Deficit Index as the decreased performance from item memory to associative memory. Back to Naveh-Benjamin, it can be said now that older adults exhibit higher ADI compared to young adults.

2. We have clarified the paragraph on page 22 lines 463-473 where we discuss possible future research. We wrote: “Previous studies that examined divided attention in younger adults, reported on minimum memory deficit during retrieval compared to encoding. Our study simulated older adults’ associative deficit by using a dual task that required WM updating only in the encoding phase. Therefore, it is important to investigate the associative-deficit in younger adults under DA conditions using dual-task at retrieval. Hence, future research needs to simulate older adults’ associative-deficit in younger adults by using a dual-task in the test phase in parallel to, and separated from, the encoding phase. It is possible that associative deficit will be obtained not only in the encoding but also in the retrieval phase and therefore will generate a larger associative deficit. Such results, if obtained, might provide more accurate understanding of older adults’ associative deficit".
