# Supplementary material for: A simulation of older adults’ associative memory deficit using structural process interference in young adults
Source: PLoS One. 2021 Nov 18;16(11):e0258574. doi: 10.1371/journal.pone.0258574 (PMC8601526; doi:10.1371/journal.pone.0258574)
Supplement: Attachment — Submitted filename: responses R2 7.7.21.docx [file pone.0258574.s005.docx]

The manuscript has now been re-reviewed by both original reviewers. While Reviewer #2 feels their concerns are now sufficiently addressed, Reviewer #1 still has substantial concerns regarding the readibility of the introduction, the statistics and figures reported in the results section, and parts of the discussion where some conclusions do not seem supported by the results. All of these points need to be thoroughly addressed in the next revision.


**Comments to the Author**

1. If the authors have adequately addressed your comments raised in a previous round of review and you feel that this manuscript is now acceptable for publication, you may indicate that here to bypass the “Comments to the Author” section, enter your conflict of interest statement in the “Confidential to Editor” section, and submit your "Accept" recommendation.

Reviewer #1: (No Response)

Reviewer #2: (No Response)

2. Is the manuscript technically sound, and do the data support the conclusions?

The manuscript must describe a technically sound piece of scientific research with data that supports the conclusions. Experiments must have been conducted rigorously, with appropriate controls, replication, and sample sizes. The conclusions must be drawn appropriately based on the data presented.

Reviewer #1: Partly

Reviewer #2: (No Response)

3. Has the statistical analysis been performed appropriately and rigorously?

Reviewer #1: No

Reviewer #2: (No Response)

4. Have the authors made all data underlying the findings in their manuscript fully available?

The [PLOS Data policy](http://www.plosone.org/static/policies.action#sharing) requires authors to make all data underlying the findings described in their manuscript fully available without restriction, with rare exception (please refer to the Data Availability Statement in the manuscript PDF file). The data should be provided as part of the manuscript or its supporting information, or deposited to a public repository. For example, in addition to summary statistics, the data points behind means, medians and variance measures should be available. If there are restrictions on publicly sharing data—e.g. participant privacy or use of data from a third party—those must be specified.

Reviewer #1: Yes

Reviewer #2: (No Response)

5. Is the manuscript presented in an intelligible fashion and written in standard English?

PLOS ONE does not copyedit accepted manuscripts, so the language in submitted articles must be clear, correct, and unambiguous. Any typographical or grammatical errors should be corrected at revision, so please note any specific errors here.

Reviewer #1: No

Reviewer #2: (No Response)

6. Review Comments to the Author

Please use the space provided to explain your answers to the questions above. You may also include additional comments for the author, including concerns about dual publication, research ethics, or publication ethics. (Please upload your review as an attachment if it exceeds 20,000 characters)

Reviewer #1: I acknowledge that the authors did a good amount of work in revising the manuscript according to the concerns that I raised. While I see an improvement in the quality of the manuscript, I still have several major concerns preventing publication.
- **The introduction** still needs to be substantially revised for the sake of clarity and smoothness. I still had trouble reading it, and I found the reading highly laborious.

The introduction has undergone significant revision and editing. We hope that it is now easier to read.

Here are a few examples of the kind of things that lack clarity, but the revision needs to be extended to the entire introduction section:
o P.2: «Various studies suggested several explanations to older adults’ memory decline: a reduction in attention, processing resources and speed [1-3], a decline in memory performance…» : the authors state that older adults’ memory decline is explained by a decline in memory performance.

Thanks. We corrected that sentence and hope it is good now.

o P. 4: «Failures to simulate older adults’ associative-deficit in younger adults stems from the secondary tasks used in the research [10, 16-17]» : it is not clear if this is a result shown in the literature, or is the hypothesis that the authors wish to test, or is it a hypothesis suggested by other authors?

We revised the whole paragraph. See pp.4-5, lines 81-89.

o P. 4: «Additionally, these results support the possible validity of an independent associative mechanism» : it is not clear what results this refers to

We revised and rewrote the paragraph that this sentence was a part of. The information is organized differently now throughout the introduction.

o P. 5: «Moreover, the secondary tasks used in the this research do involve WM, but not continuous WM. Namely, each step in the experiment is independent of the other [12].»: it is not clear why and how this is important, while it should be clear at this stage of the introduction.

Thanks for the comment. When using a secondary task, we wanted to look for a continuous resource-allocating task that would not leave spare time and attention to carry out the other memory related processes. This is now clarified in p. 5, lines 81-89.

The importance of looking for a continuous secondary task is based on previous studies:

Naveh-Benjamin, M., Craik, F.I.M., Guez, J., & Dori, H. (1998). Effects of divided attention on encoding and retrieval processes in human memory: Further support for an asymmetry. *Journal of Experimental Psychology: Learning, Memory and Cognition, 24(5),* 1091-1104.

Naveh-Benjamin, M., & Guez, J. (2000). The effects of divided attention on encoding and retrieval processes: Assessment of attentional costs and a componential analysis. *Journal of Experimental Psychology: Learning, Memory and Cognition, 26(6)*, 1461-1482.

**- Result section:**
o P. 11: Could the authors detail the effects of the interactions that are significant, using post-hocs? (attention x test & attention x task-group)

Done.

o P. 11: «Further analysis of the interaction between attention and test were significant only for the NBL task (F (1, 32) = 12.80, p < .01, η2 = .29) but not for the parity task (F (1, 32) = 2.25, p = 0.14, ns.)» : in my understanding, this is actually a further analysis of the triple interaction, which is non-significant

You are right. We present these planned comparisons, to directly test our hypothesis regarding the specific effect of divided attention using NBL task, compared to Parity task (or VS in experiment 2). We have added statistical reference to justify our choice.

o P.16: could the authors detail the direction of the significant interaction using post-hocs? (attention x test)

Done.

o Intermediate discussions p. 13 and p.18: caution in the interpretation of the results should be taken, as the triple interactions were not significant

- Overall, in the interpretation of the results in the discussion, I think caution should be taken in sentences like, e.g., «The present research succeeded in obtaining an associative-deficit in younger adults by performing a secondary task that requires updating» as the triple interactions were non-significant in both experiments

Thanks for your comment.

a. You are right and now we have refined the above conclusion, see P. 23-24 lines 470-472 "The present research suggested a new direction in understanding age associative deficit by using a structural process interference in young adults. Our current findings focus on WM updating and more studies are needed in order to establish this line of research."
b. In order to be in the "safe size" we support our analysis in methodological reference claims that significant ANOVA results are not a pre-condition for performing focused contrasts (see Rosnow & Rosenthal, 1995, p. 4). Although the three-way interaction in the full-factorial analysis was not significant, we performed our planned comparisons to test the interaction between *Group* and *Test* separately for each attention condition and obtained associative deficit.

- **Figure 2 and 3**: precise in the legend to which experiment each figure refers to. Also, in Figure 2, please homogenize the graphs so that we can visually compare performance across tasks

Done.
