# Supplementary material for: A simulation of older adults’ associative memory deficit using structural process interference in young adults
Source: PLoS One. 2021 Nov 18;16(11):e0258574. doi: 10.1371/journal.pone.0258574 (PMC8601526; doi:10.1371/journal.pone.0258574)
Supplement: Attachment — Submitted filename: response to reviewers 03.09.docx [file pone.0258574.s006.docx]

(1) There are still many grammatical errors throughout the manuscript, in particular in the introduction and abstract, which should be corrected to improve the quality and readability of the manuscript. These sections should also avoid vague language like “it seems” and “it appears”, and instead clearly state what can be inferred based on the existing literature

The manuscript was edited again by another professional editor of scientific articles.

(2) The figure legends should be expanded to specify exactly what is represented by the bar graphs and error bars (e.g., mean +/- SEM). They should also contain an explanation of the abbreviations (FA and DA) in the legends themselves. Finally, text in the figures should be corrected for typos (e.g., “visuospatial” instead of “visuospatial” in Fig. 3.

Figures were corrected.
